# Multisystem inflammatory syndrome in children associated with Bacille Calmette-Guérin scar: a case presentation

**DOI:** 10.1186/s12887-023-03911-8

**Published:** 2023-03-01

**Authors:** Hilal Kizildag, Kristin Joanna Hannavi, Rana Gur, Kazim Oztarhan

**Affiliations:** TC Demiroglu Bilim Universitesi, Istanbul, Turkey

**Keywords:** MIS-C, Kawasaki disease, BCG scar, SARS-CoV-2, PIMS-TS

## Abstract

**Background:**

During Coronavirus disease of 2019 (COVID-19) pandemic, the WHO reported a noticeable increase in Kawasaki disease prevalence in countries where Kawasaki disease is rare. This newly seen disease, unlike typical Kawasaki disease, tends to appear at a later age, has prominent gastrointestinal findings, higher rates of myocarditis and coronary artery involvement and a greater need for admission to the intensive care unit (ICU). Induration of the Bacillus Calmette-Guerin (BCG) scar is a rare finding seen in multisystem inflammatory syndrome (MIS-C). This is the second reported case of erythema and induration of the BCG scar in a 1-year-old boy with MIS-C.

**Case presentation:**

The Arabic boy presented with high resistant fever, nausea/vomiting, diarrhea, erythematous lips, and conjunctivitis. He later developed induration of his BCG scar, diffuse rash and desquamation on fingers and toes. He had a history of COVID-19 exposure as his IgG antibodies against severe acute respiratory syndrome coronavirus 2 (SARS-CoV-2) were positive. Based on his clinical findings and repeated lab results, he was diagnosed with MIS-C with Kawasaki features and treated with intravenous immune globulin (IVIG) followed by methylprenisolone and aspirin.

**Conclusions:**

Reaction at the BCG inoculation site is not a diagnostic criteria for Kawasaki, but it is seen clinically in 30-50% of the patients. We report the case of a 1-year-old boy diagnosed with MIS-C presenting with erythema and induration of BCG scar. Further studies are needed to explore this clinical presentation, especially in the countries that have BCG vaccination programs, and to determine the mechanisms of MIS-C.

## Background

Coronavirus disease (COVID-19) is an infectious disease caused by the SARS-CoV-2 virus. The clinical manifestations of novel COVID-19 vary from upper respiratory and influenza-like symptoms to serious lethal pneumonia. Infection in children appears to be more commonly transmitted from an infected family member [1]. Children with COVID-19 may be asymptomatic or have only mild respiratory and/or gastrointestinal symptoms that might be missed. Children may act as a potential source for transmission as the disease tends to be mild and may therefore go undiagnosed [2].

Kawasaki disease is a common primary vasculitis in childhood, affecting medium and small-sized arteries. The incidence of the disease is highest in Asia, especially in Japan with more than 300 cases per 100,000 children aged 4 years or younger, compared to 25 cases per 100,000 children aged 5 years or younger in North America [3, 4]. The etiology of the disease is still unknown. It often occurs after viral infections such as Enteroviruses, Adenoviruses and Rhinoviruses, with new Kawasaki-like cases being reported after SARS-COV-2 [5].

During the COVID-19 pandemic, the WHO reported that there has been a noticeable increase in the prevalence of a multisystem inflammatory condition similar to Kawasaki disease especially in countries where Kawasaki disease is rare [5]. These Kawasaki-like findings are named pediatric inflammatory multisystem syndrome temporally associated with SARS-CoV-2 infection (PIMS-TS) in Europe and multisystem inflammatory syndrome in children (MIS-C) in the United States [6, 7].

Unlike Kawasaki disease; gastrointestinal symptoms, lymphadenopathy, myocarditis, requirement of intensive care support, coronary artery involvement and intravenous immunoglobulin resistance are seen more frequently along with late-onset of age in MIS-C [8].

An important clinical sign that is not included in the classical clinical criteria for Kawasaki disease is a reaction at the Bacille Calmette-Guérin (BCG) scar. To the best of our knowledge, to date there has only been one reported case in the literature of a BCG inoculation site reaction in a patient with MIS-C in Japan [9]. Here, we report a case of MIS-C in a one-year-old boy accompanied by erythema and induration at the BCG scar in Turkey.

## Case description

The patient is a 1-year-old Arabic boy with no remarkable medical history. He was however in close contact with his parents who had symptomatic COVID-19 3 weeks prior to admission. The family were on holiday in Turkey when the patients initial symptoms occurred. He presented to the Istanbul Florence Nightingale Hospital with a 2-day history of high-grade resistant fever, nausea/vomiting, diarrhea, erythematous lip and conjunctivitis.

**Fig. 1:**
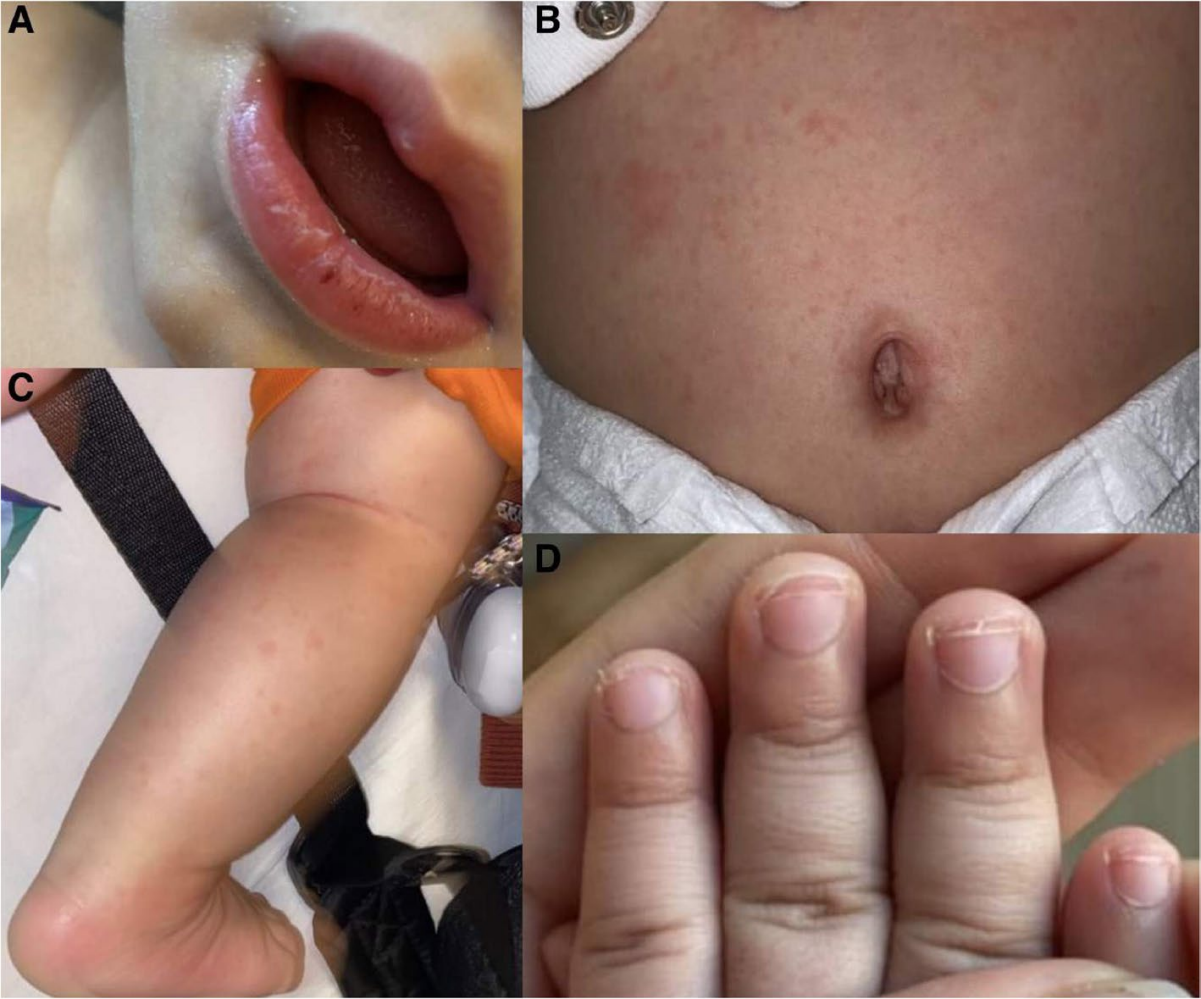
Cracked erythematous lips (**A**) diffuse maculopapular rash (**B**, **C**) desquamation on his fingers and toes (**D**)

**Fig. 2:**
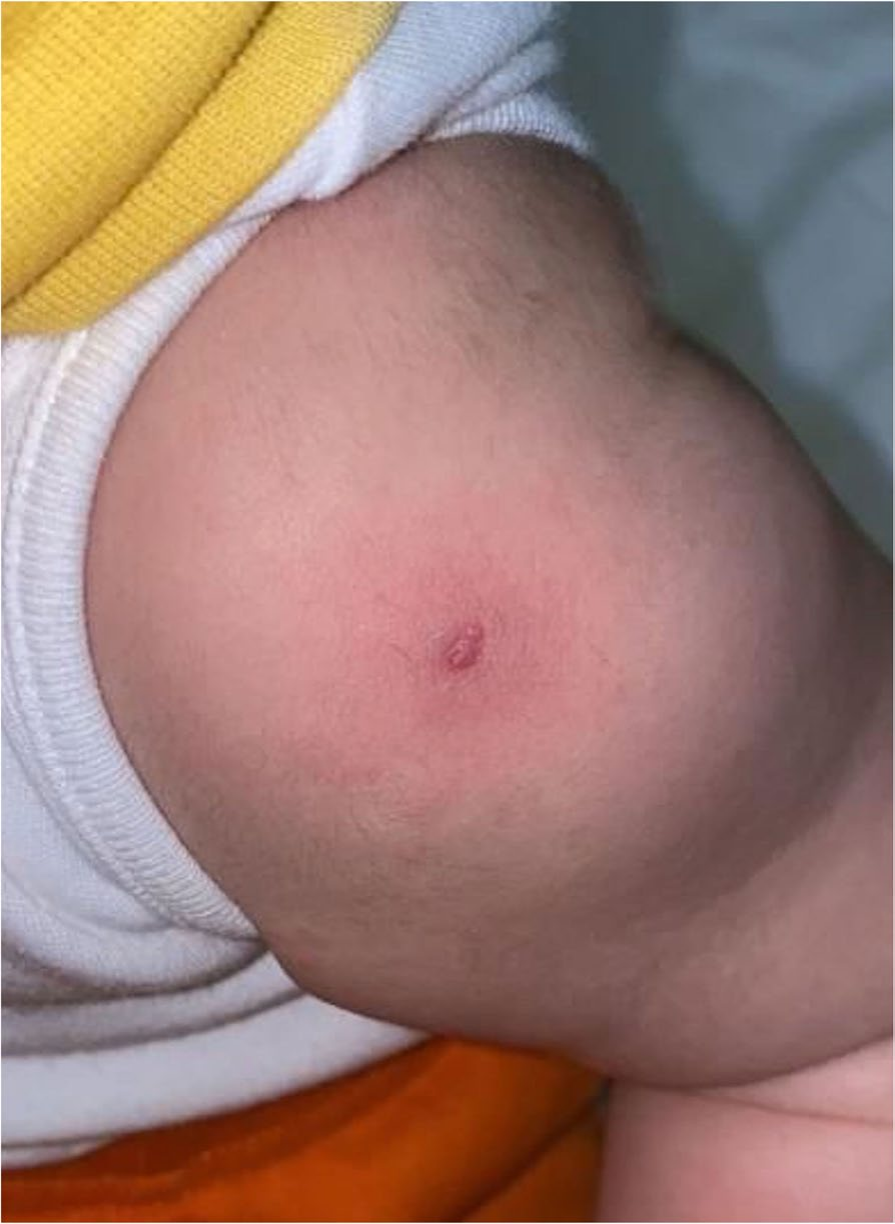
Erythematous induration of BCG scar

On examination, he was febrile with a temperature of 38.9 °C which was his highest recorded temperature during his admission. His physical exam showed mucocutaneous lesions on his tongue and cracked erythematous lips (Fig. 1A) with no cervical lymphadenopathy. He developed erythematous induration of his BCG scar (Fig. 2) on the 7th day of his hospitalization. A diffuse maculopapular rash (Fig. 1B, C) and desquamation of his fingers and toes (Fig. 1D) occurred on the 10th day of admission.

His lab results are listed in Table 1 according to the day of admission the samples were taken.

**Table 1:** Time course of laboratory findings

Day of admission	Day 1	Day 3	Day 5	Day 10	Day 15 (Discharge)
Leukocyte (× 10^3^/μL)	19.7	21.3	22.9	13.6	11.5
Neutrophil (× 10^3^/μL)	9.24	10.2	11	2.94	2.5
Lymphocyte (× 10^3^/μL)	9.31	9.11	9.37	8.58	7.6
Hematocrit (%)	30.3	30.4	27.6	27.8	28.3
Hemoglobin (g/dL)	10.1	10.2	9.29	9.02	9.04
Platelets (× 10^4^/μL)	434	442	614	1373	965
Albumin (g/dL)	4.2	4.1	3.9	3.4	3.8
Troponin I (pg/mL)	0.01	0.01	0.01	0.01	0.01
CRP (mg/L)	81.8	178.1	163.1	26.7	8.4
Aspartate aminotransferase (IU/L)	35	31	23	55	28
Alanine aminotransferase (IU/L)	10	9	6	24	19
Blood urea nitrogen (mg/dL)	5	7	7	9	7
Creatinine (mg/dL)	0.42	0.44	0.34	0.4	0.39
Sodium (mEq/L)	134	135	139	138	137
Potassium (mEq/L)	4.5	4.5	4.9	5.5	4.5
Procalcitonin (ng/mL)	0.72	2.67	2.45	0.75	0.45

On the first day of admission, his laboratory tests showed elevated CRP and WBC levels. His blood, urine and stool cultures showed no bacterial growth. The result of the COVID-19 polymerase chain reaction (PCR) was negative. He was initially administered IV hydration and ceftriaxone.

On the third day, his CRP and WBC levels increased. While his oral intake and mobility improved, he still had ongoing fever with reduced peak temperatures and frequency. He had a normal heart rhythm with a low-grade murmur. We were unable to perform echocardiography on this day as the family initially declined the procedure.

On the fifth day, CRP and hemoglobin levels slightly decreased while WBCs and thrombocyte levels increased. The family consented to echocardiography, which showed a silent PDA, no coronary dilation and normal heart contractions with 72% EF. Troponin I, BNP, and NT-proBNP levels were normal. He continued to receive antibiotic therapy as his CRP levels had started to gradually decline.

On the seventh day, he developed erythematous induration of his BCG scar along with ongoing fever. The result of repeated COVID-19 polymerase chain reaction (PCR) remained negative while serologic testing for IgG antibodies against SARS-CoV-2 was positive. His abdominal USG and chest x-ray showed no abnormalities. Repeated echocardiography showed no new findings. Based on his persistent fever along with other clinical symptoms, signs and laboratory results, a diagnosis of MIS-C was made. Antibiotic therapy was discontinued and IVIG therapy was recommended but initially declined by the family.

On the eighth day of admission, the family consented for the patient to receive IVIG at 2 g/kg as a single infusion given over 10 hours. His fever along with other clinical symptoms remained persistent unlike we expected. Planned second dose was declined by the family.

On the ninth day, he was switched to 2 mg/kg/day methylprednisolone and 80 mg/kg anti-inflammatory aspirin therapy. Forty-eight h after combination therapy, his fever subsided along with visible fading of the BCG scar which then fully disappeared on the 14th day. Methylprednisolone and aspirin therapy were discontinued on the 14th day of admission following resolution of his symptoms. He was discharged on the 15th day of admission on 5 mg/kg/day antiaggregant aspirin therapy for 6 weeks. The patient had an uneventful follow-up at an outpatient clinic on return to his home country. We scheduled a follow-up appointment in our clinic 3 months after his discharge.

## Discussion and conclusions

Kawasaki-like findings seen after SARS-CoV-2 infection are named as pediatric inflammatory multisystem syndrome temporally associated with SARS-CoV-2 infection (PIMS-TS) in Europe and multisystem inflammatory syndrome in children (MIS-C) in the United States [6, 7]. Children with Covid-19 infection frequently present with milder symptoms and are less commonly tested than adults. As a result, the exact incidence of MIS-C among children infected with SARS-CoV-2 is unclear. It however appears to be a relatively rare complication of COVID-19 in children, occurring in < 1% of children with confirmed SARS-CoV-2 infection [10].

As this case meets the diagnostic criteria, it can be described as Kawasaki disease with a history of coincidental SARS-CoV2 infection. However, MIS-C was suspected due to persistent fever, prominent gastrointestinal symptoms and SARS-CoV-2 IgG positivity. Treatment was therefore planned and administered accordingly but there were some challenges in obtaining parental consent for certain procedures and recommended treatments. The diagnosis was confirmed when the induration of the BCG vaccine became prominent and desquamation of fingers and toes appeared. Reaction at the BCG scar is not a diagnostic criterion for Kawasaki, but it is seen clinically in 30-50% of the patients [11]. To our knowledge there has only been one report of a BCG scar reaction in a Hispanic patient with MIS-C in Japan. Our patient is a 1-year-old Arabic boy, the second reported case of BCG scar reaction associated with MIS-C along with Kawasaki features [9].

Although cases of MIS-C are more likely to seen in older children, 20% of reported cases occur in children aged 1-4 years old as seen in our patient [12]. Other diagnoses were ruled out based on a detailed history, repeated medical examinations and lab results.

The most serious complication of Kawasaki disease is development of coronary artery lesions (CALs), which occurs in 15 to 25% of untreated patients. Identifying the risk factors that increase the possibility of developing CALs is important. Researchers have developed a number of scoring systems over time to evaluate the risk of complications in patients with Kawasaki disease. The Harada score (HS) is one of the commonly used scoring systems. HS scores (0 to 7 points) includes the presence of white blood cell count > 12,000/mm^3^ (1 point), platelet count < 350.000/mm^3^ (1 point), CRP > 3 mg/dL (1 point), hematocrit < 35% (1 point), albumin < 3.5 g/dL (1 point), age ≤ 12 months (1 point), and male sex (1 point) with cutoff value ≥4. Our patient had a Harada score of 5, suggesting an increased risk of coronary artery involvement. Despite his raised Harada score, coronary artery involvement did not occur. Because the Harada score was developed in Asian populations, the sensitivity and specificity for predicting coronary artery aneuryms in non-Asian populations may not be the same as in Asian populations [13].

Although the mechanisms involved are not fully understood, erythema and induration of the BCG scar has been linked to viral infections such as measles [14] as well as vaccinations, particularly influenza [15] and SARS-CoV-2 [16]. Immunological mechanisms such as delayed hypersensitivity reactions associated with T cells, interleukin-1β, and tumor necrosis factor-α [4], as well as antigen cross-reactivity between mycobacterial heat shock protein (HSP) 65 and human homolog HSP 63, which are antigenic proteins in BCG bacteria [17], have been suggested as part of the pathogenesis of erythema and induration of the BCG scar in Kawasaki disease [9].

According to various reports, there is discussion over the BCG vaccine’s ability to protect against COVID-19. Though the BCG vaccination was originally introduced to prevent tuberculosis, more recent studies have shown that the BCG vaccine has nonspecific benefits in enhancing innate immunity by endowing innate cells with an immunological memory. It has been demonstrated that this “trained innate immunity” protects against some viral infections [5]. Several cases of BCG scar reaction following SARS-CoV-2 immunization have recently been reported [16, 18]. The envelope protein of SARS-CoV-2 shares a significant similarity with the Mycobacterium antigen, and BCG immunization induces specific immunity against SARS-CoV-2 [9]. The erythema and induration of the BCG scar in MIS-C patients with Kawasaki features may shed light on the syndrome’s etiology.

This MIS-C case of a 1-year-old Arabic boy with erythema and induration of BCG scar showing Kawasaki features highlights that BCG scar changes after SARS-CoV-2 infection may be characteristic of MIS-C. BCG scar changes, which were observed on the 7th day of admission in our case, are noteworthy as the history of COVID-19 infection may be missed in children with mild symptoms. To determine the mechanisms of BCG scar changes and its connection with MIS-C, further studies are needed to explore, especially in the countries that have BCG vaccination programs.

## Data Availability

All Data and material collected during this study are available from the corresponding author upon reasonable request.

## Abbreviations

COVID19: Coronavirus disease of 2019
WHO: World Health Organization
MIS-C: Multisystem inflammatory syndrome
BCG: Bacillus Calmette-Guerin
SARS-CoV-2: Severe acute respiratory syndrome coronavirus 2
LAP: Lymphadenopathy
CRP: C-reactive protein
WBC: White blood cell count
PDA: Patent ductus arteriosus
EF: Ejection fraction BNP brain natriuretic peptide
NT-proBNP: N-terminal prohormone of brain natriuretic peptide
IVIG: Intravenous immune globulin
HS: Harada Score
HSP: Heat shock protein
ICU: Intensive care unit
